# The Impact of Armed Conflict on the Prevalence and Transmission Dynamics of HIV Infection in Libya

**DOI:** 10.3389/fpubh.2022.779778

**Published:** 2022-03-31

**Authors:** Mohamed Ali Daw, Abdallah Hussean El-Bouzedi, Mohamed Omar Ahmed

**Affiliations:** ^1^Department of Medical Microbiology and Immunology, Faculty of Medicine, University of Tripoli, Tripoli, Libya; ^2^Department of Statistics, Faculty of Science, University of Tripoli, Tripoli, Libya; ^3^Department of Microbiology and Parasitology, Faculty of Veterinary Medicine, University of Tripoli, Tripoli, Libya

**Keywords:** HIV/AIDS, armed conflict, Libya, transmission dynamics, HIV-migration

## Abstract

The interrelationships between HIV/AIDS and armed conflict are a complex phenomenon, and studies are rarely devoted to this area of research. Libya is the second-largest country in Africa that has been evoked with war since the NATO intervention in 2011. The country has also experienced one of the largest HIV outbreaks associated with the Bulgarian nurse's saga. The effect of the armed conflict on the dynamic spread of HIV is not yet well-known. The objectives of this study were to determine the impact of armed conflict on the epidemiological situation of HIV infection in Libya and to analyze the transmission dynamics of HIV strains during the conflict. We investigated the movement of people with HIV during the Libyan armed conflict, analyzed the HIV subtypes reported from 2011 to 2020, and followed up the infected cases all over the country. The patterns of HIV spread within the Libyan regions were traced, and the risk factors were determined during the conflict period. A total of 4,539 patients with HIV/AIDS were studied from the four regions during the Libyan conflict. Our data analysis indicated that Benghazi, the biggest city in the Eastern region, was the significant exporter of the virus to the rest of the country. The viral dissemination changes were observed within the country, particularly after 2015. A major virus flows from the Eastern region during the armed conflict associated with internally displaced people. This resulted in the dissemination of new HIV strains and accumulations of HIV cases in western and middle regions. Although, there were no significant changes in the national prevalence of HIV/AIDS. Our data highlight the factors that complicated the spread and dissemination of HIV during the armed conflict, which provide a better understanding of the interaction between them. This could be used to plan for effective preventive measures in tackling the spread of HIV in conflict and post-conflict settings.

## Background

Human Immune Deficiency Virus (HIV) remains to be a serious problem, particularly within developing countries. Many of the countries affected by HIV are also ravaged by decades of wars and political instability. The interrelationships between armed conflicts and infectious diseases have been considered inconsistent, and studies are rarely devoted to these phenomena (1). The armed conflicts and diseases usually link to one another in a positive direction, with violent conflict driving the incidence and prevalence of disease(s) upward. Infectious diseases vary greatly in transmissibility during armed conflicts. Airborne and fecal-oral pathogens, such as tuberculosis, cholera, influenza, severe acute respiratory syndrome coronavirus 2 (SARS-CoV 2), coronavirus disease 2019 (COVID-19) pandemic, smallpox, typhus, dengue fever, malaria, plague, and yellow fever, can spread easily in conflict situations due to poor sanitation and population movements or refugee flows (1, 2). This, however, is not the case for HIV which requires quite specific conditions for transmission. Therefore, studies are needed to highlight the impact of armed conflict on the epidemiological situation of HIV in war-torn countries (3).

The Northern African region continues to account for the large majority of the global HIV/AIDS reported cases in the last decade and it was one of the regions that have been more acutely affected by large-scale violent conflicts than other regions in the world (4). Interestingly, no studies have been published about the dynamics of the relationship between the two crises. However, epidemiological studies in this area of research are limited and even indicate an ambiguous and complex relationship between conflicts and HIV prevalence levels worldwide.

In Ukraine, which has one of the largest HIV epidemics in Europe, the war in the east of the country had reignited the virus spread and studies suggest that effective prevention responses should involve internally displaced people and people who frequently travel to war-affected regions (5).

Studies from sub-Saharan Africa (SSA) showed that at the end of the Angola conflict in 2002, the country's HIV prevalence was relatively lower than the rates in other Southern African countries, which suggests that conflicts may have slowed HIV spread in this case. However, in Guinea-Bissau, which experienced a civil war in 1998–1999, the civil war in 1998–1999 may have sparked HIV-1 transmission, as HIV-1's prevalence more than doubled between 1997 and 1999, but there is no evidence of a long-term effect on the trends of HIV-1 or HIV-2 prevalence. Hence, further studies are needed to highlight the dual-burden of violent conflict and infectious disease (6–8).

Libya, the second-largest country in Africa with the longest coast in the Mediterranean basin, has been evoked in a major destructive armed conflict since 2011. During the conflict, health care services were continuously interrupted and the conflict caused massive internal population displacement. Over 1.5 million people have been internally displaced in Libya out of a total population of 6 million (7–11). Furthermore, the country has experienced one of the largest HIV/AIDS outbreaks associated with the Bulgarian nurse's saga in Benghazi in the Eastern region of the country (12, 13), suggesting that parenteral transmission played a role in the spread of HIV in the early epidemic. Since then, Libya, in cooperation with the European Union, has released the Bulgarian nurses and scaled up highly efficient scientific research programs to treat the infected victims, introduce a harm-reduction approach, and trace the infected individuals (14, 15). The program was found to be successful and has been shown to reduce risky practices and HIV transmission among the Libyan population. A community-based study in the country points out that the number of newly registered HIV cases dropped immensely in Libya; the HIV prevalence in the general population stands at >0.05%, thus, indicating that the epidemiological situation had stabilized (16). This epidemiological stability was disrupted by internal protest in 2011 in the Eastern region complicated by the North Atlantic Treaty Organization (NATO) military intervention and a continuous civil war until October 2020 (17, 18).

Human migration and population displacement are likely to have an impact on epidemic dynamics within and among the Libyan regions. As the people infected with HIV move to new places, they might disseminate new viral strains. In addition, new groups of susceptible individuals may be generated, affected by war, with potentially poor access to health care. Hence, increased export of HIV from the Eastern region (most heavily HIV-affected region and where the war escalation started) to other Libyan regions are expected (19, 20). The dual burdens of HIV/AIDS and armed conflict will be a major obstacle to the development of a country. However, little is known about the dynamics of the relationship between the two crises in Libya. Hence, a clearer understanding of the dynamics of the interface between conflict and HIV is crucial for the development of effective and efficient strategies to reduce population risk. The objectives of this study were to determine the impact of armed conflict on the epidemiological situation of HIV-1 infection and analyze the changes in the prevalence and dynamics of HIV-1 subtypes overtime during the conflict period and through all Libyan geographical regions.

## Materials and Methods

### Search Strategy and Data Collection

This research involved a comprehensive analysis of the impact of the Libyan armed conflict on the epidemiological situation of HIV-1 infection during the conflict period from 2011 to 2020. The research strategy was based on analyzing the accurate data collected during the conflict period, We used all the data collected from the four Libyan regions (East, West, North, and South) available from the Libyan National HIV database. Professional epidemiologists collected and analyzed data.

In Libya, the HIV surveillance system is based on mandatory and anonymous notification of newly diagnosed HIV cases by laboratories all over the country, combined with epidemiological information on the mode of transmission and other clinical data, as reported by physicians and trained clinical epidemiologists. All laboratory tests were carried out and confirmed at the Libyan central laboratory –Tripoli using international standard tests for HIV detection and subtyping. More information on sampling, phylogenetic analysis, and reference dataset was previously reported (4, 16). The data collected from all over Libyan regions of persons infected with HIV during the Libyan conflict from 2011 to 2020 are shown in Table 1.

**Table 1:** Number of reportedly HIV-infected people during the Libyan armed conflict in ten years (2011-2020).

**Year**	No. Cases^*^	**Male**	**Female**	**Ratio (M:F)**	**Prevalence rate/100,000 people**
2011	371	293	78	4:1	5.3
2012	486	385	101	4:1	6.9
2013	459	338	121	3:1	6.9
2014	439	359	80	4:1	6.3
2015	511	372	139	3:1	7.3
2016	309	212	97	2:1	4.4
2017	471	382	89	4:1	6.7
2018	492	387	105	3:1	7.0
2019	489	382	107	4:1	7.0
2020	512	399	113	4:1	7.3
Total	4,539	3,509	1,030	3:1	64.84

Number of seroincident individuals^*^.

### Demographic Factors

The demographic data included were collected for all patients, including gender (male/female), behavioral data and transmission risk factors, people who inject drugs (PWID), heterosexuals, others, residential area (region, province postcode), resident or displaced, and age category (<20,20–29, 30–39, 40–49, and ≥50).

### Tracing and Migration Pathway

A comprehensive and detailed follow-up of each patient infected with HIV was carried out during the ten years of the Libyan conflict from 2011 to 2020. This consists of patient location, HIV conditions, and the transmission dynamics. The viral gene flow was indicated as the number of migration events; this is the number of viral strain movements from one location (i.e., region) to another.

The numbers of HIV-1 subtype migration exportation and importation events were correlated with the epidemiological characteristics of HIV within each region. This includes all the Libyan dataset's patients with HIV. Epidemiologic data were gained by reconstruction of the migration route.

For each region (West, Central, South, and East), we looked at the following:

- The numbers of exportation viral strain migration events from one region to all of the other regions.
- The numbers of importation viral strain migration events from one region to all of the other regions.

### Geographic and Statistical Analysis

The viral geographic transition flow that might be associated with the military conflict in the East, where the conflict started, was traced all over the country and all cases were officially according to the national case report as previously described (4). The prevalence was determined as previously described by the number of HIV confirmed cases per 100,000 (prevalence rate/100,000 people). The association of the demographic data with the HIV subtype was performed in R v3.6.2 (21) using the gplots and corrplots packages (22, 23); other related coincident were calculated in MATLAB® v2020a and regarded as significant with *p***-**values <0.05 (24).

### Ethical Approval

Routinely collected sequence and demographic data on all newly notified HIV-1 infections are linked and irreversibly de-identified to enable public health research in the country, as previously described (14, 16). The study was approved by the Libyan National Ethical Committee (Approval No. LY NS, HIV473221). It was conducted under the Helsinki Declaration and the supervision of the Libyan Study Group of Hepatitis and HIV (25, 26).

## Results

A total of 4,539 different strains of HIV were available from the Libyan HIV database. These data were collected from all over the Libyan regions (West, East. Middle, and South) within ten years from the start of the armed conflict in 2011 until 2020. Of these reported strains, 3,509 (77.3%) were reported from male and 1,030 (22.7%) in female (M: F ratio 3:1). The number of reported cases varied from 1 year to another during the study period. They were increased from 371 in 2011, to reach up to 512 in 2020, and the reported incidence rate (IR) (number of reported cases/population) rose from 6.0:100,000 in 2011, to reach up to 9.0:100,000 in 2020, as shown in Table 1.

The demographic, clinical, and HIV-1 sequences data of the enrolled participants were shown in Table 2. The median age of the participants was 37 years [inter-quartile range (IQR) of 26–49 years]. Of the 4,539 participants involved in the study, 1,369 (30.2%) were from the Eastern region, 1,685 (37.1%) are from the Western region, 937 (20.6%) are from the Middle region, and 548 (12.1%) are from the Southern region. A total of 3,070 (67.4%) were reported among the resident individuals and 1,469 (32.4%) from the displaced population. There was no variation in the number of reported cases within the resident individuals. It was found to be 16 % in 2011 and 17.2 % in 2020 (*p*-values > 0.05), while those reported from displaced individuals increased steadily from 2.9% in 2011 to 4.7 (2013–14), 12.6, and 7.2 %, and then, declined to 4.8 % at the end of the conflict period (*p* < 0.05).

**Table 2:** Demographic characteristics of HIV infected population during the Libyan armed conflict of 2011-2020.

**Study period**						
	**2011–2012**	**2013–2014**	**2015–2016**	**2017–2018**	**2019–2020**	**Total (%)**
**No of cases**
	857	898	820	963	1,001	4,539
**Study location (Region)**
East	379	394	198	178	219	1,369 (30.2)
West	237	279	397	421	351	1,685 (37.1)
Meddle	129	126	131	262	289	937 (20.6)
South	112	99	94	101	142	548 (12.1)
**Movement status**
Resident	726	681	247	634	782	3,070 (66)
Displaced	131	217	573	329	219	1,469 (34)
**Age category**
<20	93	71	47	43	67	321 (7.0)
20–29	246	194	125	127	287	979 (21.3)
30–39	523	471	311	205	582	2,092 (45.5)
40–49	230	181	117	198	291	1,017 (22.1)
>50	73	32	21	27	31	184 (4.0)
**Transmission risk factor**
IDUs	421	379	357	428	491	2,076 (45.2)
Sexual activity	209	228	231	287	301	1,256 (27.3)
Others/Unknown	227	291	232	248	209	1,207 (26.3)
Total	857	898	820	963	1,001	4,539
**HIV-1 subtype**	1,469				
A	201	195	159	218	311	1,084 (23.9)
B	370	414	365	421	490	2,060 (45.4)
CRFo2_AG	197	202	193	216	102	910 (20.1)
NI^*^	89	87	103	108	98	485 (10.7)

NI^*^, Non-Identifiable.

The majority of the study participants attributed to injecting drug users (IDUs) accounted for 2,076 (44.7%, *p* < 0.05), followed by those with a high-risk sexual behavior 1,256 (27.7%), and 1,207(26.6%) with other risk factors. No significant changes were found for transmission risk factors during the investigation period 2011-2020 (*p* > 0.05). The proportion of IDUs cases was reported to be (9.3%) in 2011, (10.8%) in 2020, (5 to 6.6%) for sexual contacts, and 6.4% for other risk factors.

The overall HIV subtype distribution was (A) 1,084 (23.9%), (B) 2,060(45.4%), CRF02_AG 910 (20.1%), and others 485 (10.7%). The trends of HIV subtypes were changed overtime during the conflict period. There is a substantial difference in the emergence of each subtype during the ten years. We observed an increase in the proportion of subtype B infections from 8.2 to 10.8%, and subtype A from 4.4 to 6.7%. For CRF02_AG, we found a significant decrease over time as it varied from 4.3 to 2.2% during 2011–2020.

**Figure 1:**
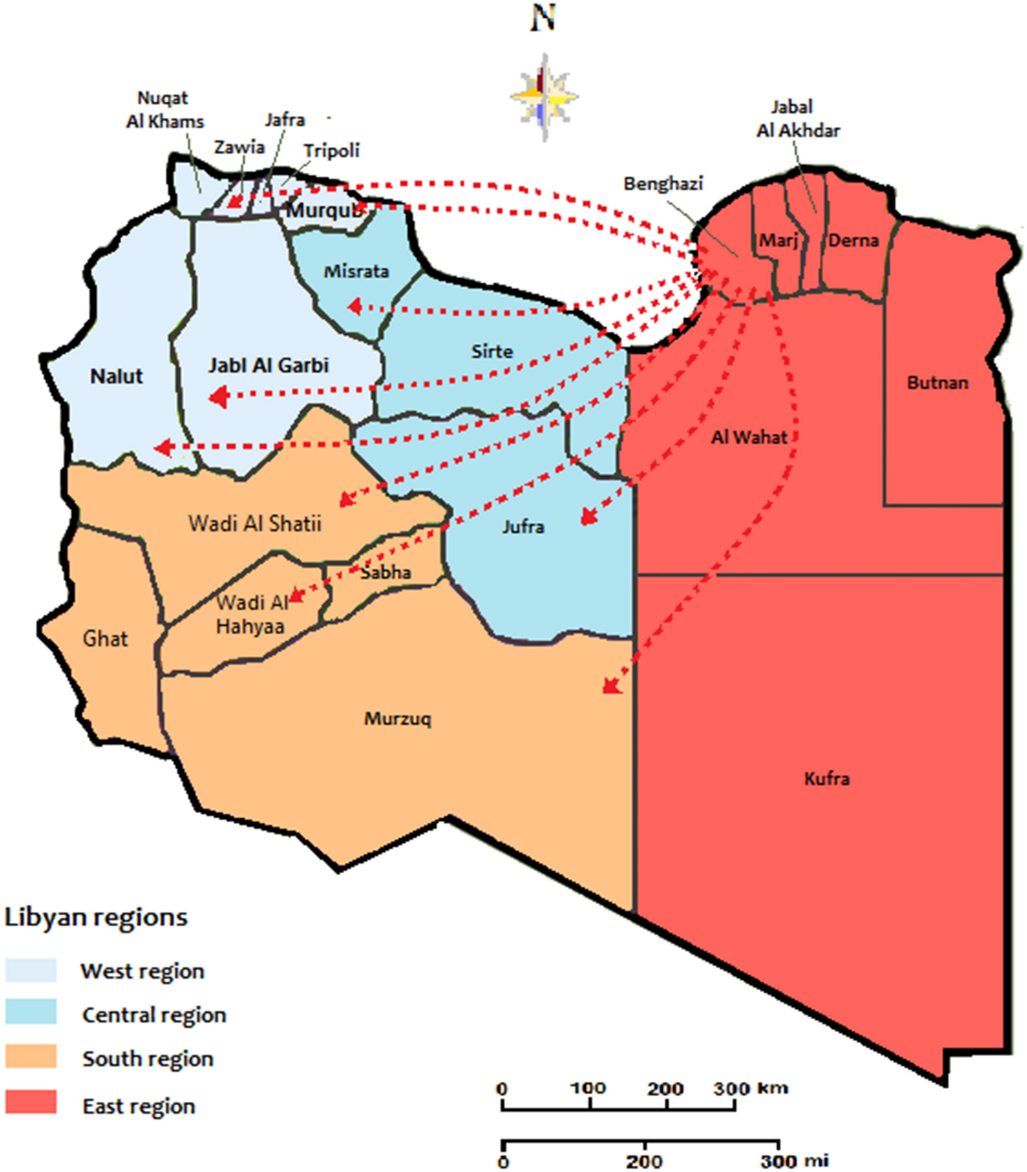
The migration and expansion patterns of HIV cases during the conflict time of 2011-2020. The arrows indicate the directionality of virus flow movement from the residential location.

The transmission dynamics of HIV Type 1 in the Libyan population during the armed conflict was illustrated in Figure 1. The country is classified into twenty-two provinces within the four national regions, the West region (7 provinces), Central region (3 provinces), South region (5 provinces), and East region (7 provinces). According to the geographic dataset analysis, the East region was the main exporter accounting for an average of 93.5% of the migration events in Libya. The geographic strains movements were mostly observed in this region. The most strongly supported viral migration route was found between Benghazi and Tripoli and Benghazi to Misrata. Other routes connecting Benghazi to Sebha and Western Mountains were frequently reported. Other routes of Genetic flow connected Tripoli to the Western mountain, Misrata to Tripoli, and Misrata to Sebha was also reported.

Table 3 illustrates the different HIV-1 strains that migrated from and to different regions during the armed conflict. Of a total of 972 type-able strains, 504 (51.9%) were migrated all over the country. Of these, 471 (48.5%, *p* < 0.05) migrated from the Eastern Region, including 321(33.0%, *p* < 0.05) recombinant Circulating recombinant strain of HIV 1 (CRF02_AG), followed by B 143 (14.7%), and only 7 (0.7%) HIV-1 A. The middle region received 17 (1.7%) migrated stains (9 CRF02_AG, 6A 2B), followed by the Western Region with 9 (0.9%) strains, and only 7 (0.7%) from the Southern Region.

**Table 3:** Inter-location series of HIV-1 strains in Libya during the armed conflict of 2011-2010.

**No cases(%)**	**Migrated to (%)**				**Migrated from (%)**			
**Location**	**Total**	**A**	**B**	**CRF02_AG**	**Total**	**A**	**B**	**CRF02_AG**
**West region**
Tripoli	319	13	224	82	9	1	5	3
**Meddle region**
Misrata	107	09	75	23	17	2	06	09
**East region**
Benghazi	39	07	19	13	471	7	143	321
**South region**
Sebha	03	0	02	01	07	4	3	0

Furthermore, 468 (48.1%) strains were migrated to different regions, including 319 (32.8 %, *p* < 0.05), which migrated to the western region, particularly Tripoli, including B 224 (23%), C 82 (8.4%), and A13 (1.311%), followed by the Middle region 107(%), A 9(9%), B 75 (7.7%), and C 23 (2.4%). Although, there are only 39 (4%) and 3 Strains migrated to eastern and southern regions, respectively, (*p* > 0.05).

**Figure 2:**
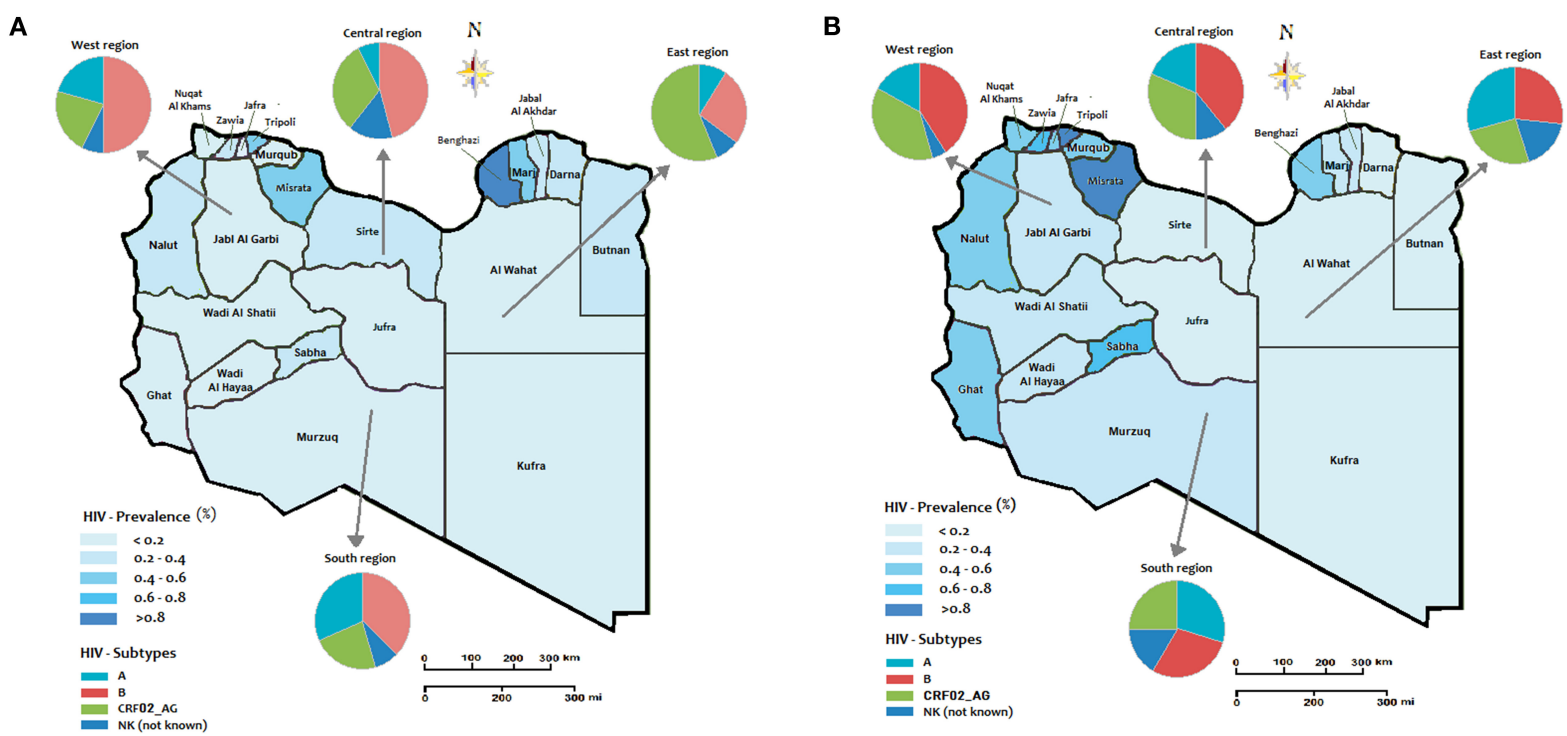
The regional prevalence of HIV and geographic distribution of HIV-1 subtypes during the Libyan conflict **(A)** the period from 2011 to 2015; **(B)** period from 2016 to 2020.

The prevalence of HIV and the distribution of multiple HIV subtypes in different geographic locations during the Libyan conflict were presented in Figure 2. In the first five years (2011-2015-Figure 2A), the Eastern region showed the highest prevalence of HIV, particularly in Benghazi, estimated to be over 0.8%, followed by Marj, Darna, and Butnan, and to a less extent, Al Wahat and Kufra. In these provinces, CRF02_AG was the predominant circulating strain accounting for 60% (*p* < 0.05), followed by Strain B with 20%. In the West region, Tripoli reported an HIV prevalence of 6% followed by Nalut's 4%, and others are <0.2%. In the Western provinces, HIV-B was the predominant strain which has reached 50%, followed by HIV-A and CRF02_AG, which accounted for 20% each. In Central province, the HIV prevalence was reported to be 0.6% in Misrata, followed by Sirte and Jufra. The HIV–B and CRF02_AG were the predominant circulating strains, where they occupied 45 and 25%, respectively. In the South region, the HIV prevalence was reported to be very low (<0.4%). The predominant circulating strains in these provinces were B (40%,), A (30%), and to a less extent, CRF02_AG (20%).

**Figure 3:**
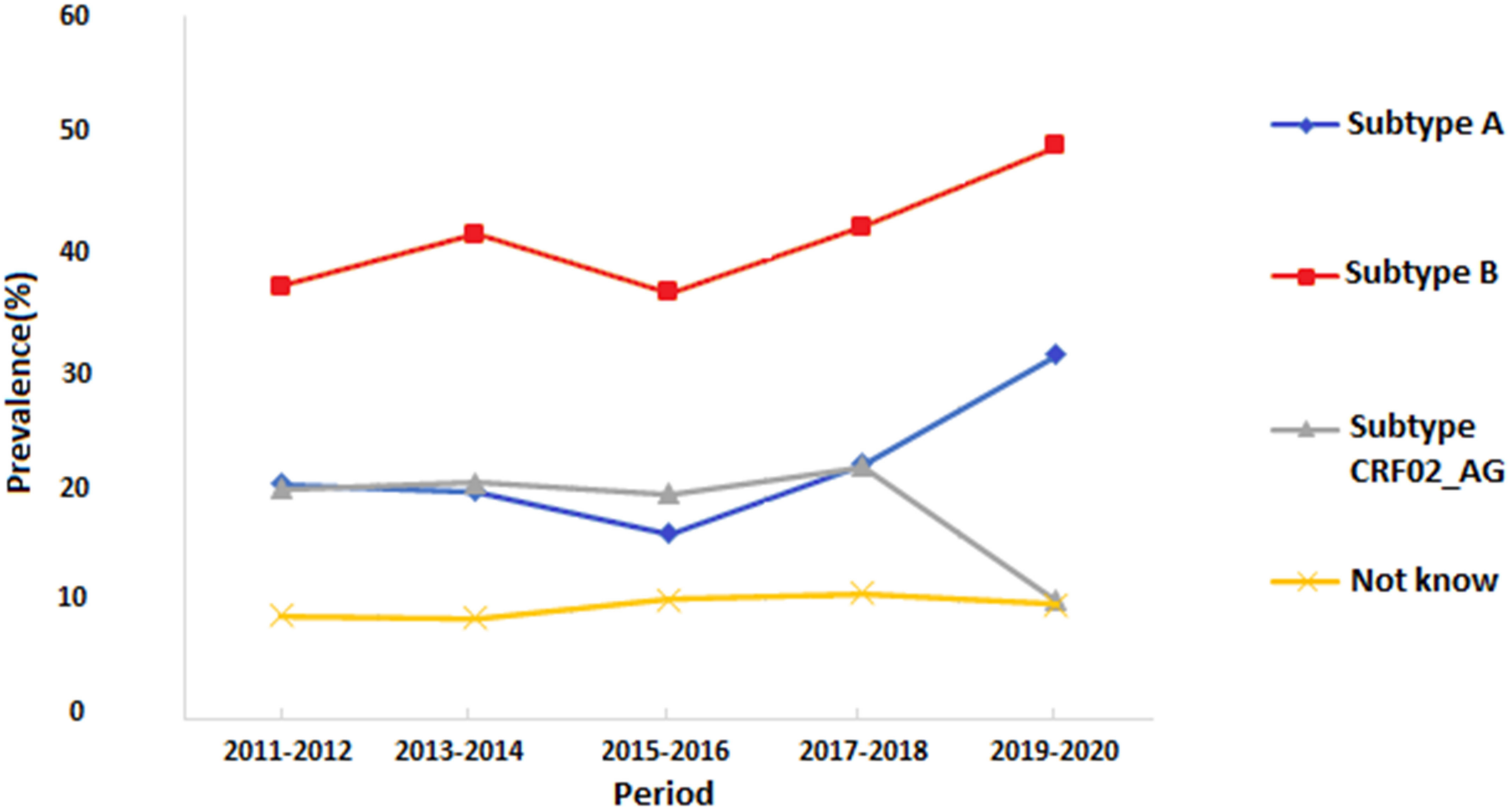
Trends of the prevalence of different HIV-1 subtypes in Libya during the armed conflict of 2011-2020.

In the second period of the conflict (2016–2020) shown in Figure 2B, the Western region showed a higher HIV prevalence. The highest prevalence was reported in Tripoli, Zawia, Nuqat Al-Khams, Jafra, Murqub, Nalut, and to a less extent, Jabl-Al Garbi. In these provinces, the highest-circulating HIV strains were B (40%), CRF02_AG (35%), and A (20%). In the Central region, the highest prevalence was reported in Misrata (>0.8%) and only <0.2% in Sirte and Jufra. HIV-strain B accounted for 40% of the circulating strains followed by CRF02_AG (35%) and B (20%). In the Southern provinces, the HIV prevalence reached 0.6% in Sabha and Ghat and 4% in the other three provinces. The most circulating strains that occupy the Southern provinces were Strains A and B, which accounted for 30% each, followed by CRF02_AG (25%). In the Eastern region, the HIV prevalence was 0.6% in Benghazi and <0.2 in the other provinces. Furthermore, the most predominant circulating strains were B (25%), CRF02_AG (25%), and A (20%). The prevalence of Such circulating strains vary from one year to another during the armed conflict as shown in Figure 3.

## Discussion

Armed conflicts may have profound effects on the spread and dissemination of the HIV pandemic, which should be considered. A long period of conflicts has resulted in greater naïveté regarding the epidemiology and prevention of the disease in sub-Saharan and North African countries (6, 27, 28). In this study, we investigated the dissemination and spread of HIV during the armed conflict in Libya by analyzing the spatial and temporal patterns of the viral flow during the ten years of the conflict. Our data indicated that HIV-reported strains have moved from the regions involved in the armed conflict, to the rest of the country. The Eastern region was the top exporter of HIV strains, as it appears that the strains were originated directly or indirectly from Benghazi; the largest city in the East region. The east location is heavily affected by HIV before and at an early stage of the armed conflict. From 2011 to 2014, a total of 1,755 cases were reported in the eastern region, accounting for 38.7% of all nationally registered cases of HIV infection.

The demographic analysis in this study indicated that the highest HIV prevalence was reported among IDUs accounting for 2,076 (45.2%) cases, followed by sexual activities 1,256 (27.7%) cases. This was mainly common among the middle age group of 30–39 years, followed by 40–49, though, it was less among other age groups. The genetic analysis has shown that HIV-1 subtype A accounted for 2,060 (45.4 %) cases, followed by HIV-1 subtype A 1,084 (23.9%), and to a less extent, CRF02_AG, for 910 (20.1%). As IDUs are the main contributing factor among internal war displacement in the study, this may create a favorable environment for new HIV transmission and facilitate the generalization of the HIV epidemic in Libya. Similar studies on HIV in conflict-affected settings in Africa suggest that regions that have received new imported HIV strains also have appropriate conditions for those strains to flourish (29, 30). In certain African countries, such as Uganda and Nigeria, the interface of culture and insecurity has played another disseminating factor of HIV infection in displacing communities. Hence, the further study should be carried out as the conflict settles down in Libya (31, 32).

The prevalence of HIV and the spread of HIV-1 subtypes have experienced tremendous changes during the Libyan armed conflict. Our study has shown the inter-location of HIV strains within the Libyan regions is an emerging phenomenon. The number of viral exportation correlates well with the number HIV infected patients in each region. We have observed that more infected people moved from the locations from where the virus is originating. This is evident in our study as more HIV-infected individuals moved from the main exporting locations (this is Benghazi at Eastern region) to the main importing locations, particularly at the Western region (i.e., Tripoli) and Middle region (Musrta). This inter-location has resulted in a major change in the prevalence of HIV infection and the dissemination of HIV strains within the Libyan region. Before and during the early stage of the conflict, the HIV prevalence was similar among most of the Libyan regions, while it has been geographically inter-located since then. In the Eastern region, where the conflict has exsanguinated, it was changed drastically from 0.6–0.8% (2011–2015) to 0.2-0.4% (2016-2020). Although, the western and Middle region escalated from 0.2–0.6% (2011–2015) to 0.4–0 >0.8 during 2016–2020. There was no significant change in the national prevalence of HIV and HIV strains during the conflict period (2011–2020).

Population displacement is a major consequence of the armed conflict, which reflects greatly on the health of the displaced people. In Libya, over 250,000 people were displaced in 2011, and this was further increased as the armed conflict escalated from 2014 to 2020. This could be reflected in the epidemiological patterns of microbial disease and the health conditions of the displaced people, including the individuals infected with HIV (33, 34). In this study, the major transition patterns of the viral strain's movements during the armed conflict have been reported, although, there is no significant change in the types of the HIV strains reported before and during the conflict period. The HIV-subtype CRF02_AG, the main strain resident in the East region and rarely reported in the other Libya regions before 2011, accounted for 68% of the migrated strains to the other regions particularly the west and central regions. The number of the exported strains correlated well with the number of registered HIV cases in each region. More infected people have moved away from the conflict zone to a more secured area. This reflects the redistribution of pre-existing infections within the country. Many residents who were initially infected in the Eastern region have moved to the Central and Middle regions due to war, resulting in the redistribution of HIV viral strains within the country. This is in concordance with other data reported from other countries inflated by war, such as Uganda and Ukraine (35, 36). Furthermore, a population displacement may be reflected in the treatment of HIV-infected individuals as patients who had to relocate because of the conflict may be more likely to reduce treatment adherence. Hence, the spread of HIV-1 strains is becoming a serious public health challenge that requires further studies to deeply understand their origin and distribution (37).

Although many studies have been conducted to determine the impact of armed conflict on infectious diseases, this study is to be considered as the first study to describe the impact of war conflict on the trans-dynamics of HIV in Middle-East and North Africa; a region that is evoked by conflicts for a long time (38, 39). Armed conflicts, however, have a great influence on the burden of infectious diseases globally. These diseases increase in wars and armed conflicts due to disruption to surveillance and response systems that were often poorly developed, to begin with. Furthermore, this prevents access to healthcare for both conflict-affected and ordinary emergencies. The proportional impact of war conflict on the epidemiological aspects of infectious diseases varies greatly around the world and vary from one disease to another; in African refugee camps, the incidence of malaria and water-borne infections was found to be higher than in Asian camps, although pneumonia and diarrhea rates were higher in Asian camps than in Africa (40, 41).

Hence, further studies are needed at the post-conflict period to improve the accuracy of the number of HIV migrant strains that fit the population distribution with the Libyan regions. However, this study clearly indicated that human migration dynamics may play a key role in the dissemination of new viral strains and influenced the risk of HIV. This may exacerbate vulnerability and accelerate the spread of HIV in post-conflict settings. Such juxtaposition, which is evident in this study, has already been reported to some extent in certain African countries including Mozambique (35). Unfortunately, the international community that intervened in 2011 and initiated this armed conflict has compartmentalized its responses to HIV and conflict in Libya. Therefore, the efforts should be combined and studies are needed to formulate long-run policies to preclude an integrated and aggressive attack on HIV in Libya.

## Conclusions and Intervention Policies

This is the first study to investigate the impacts of the armed conflict on the epidemiological patterns of HIV infection in Libya using spatial and temporal analysis. Our findings on the initial spread of HIV infections and HIV strains provide quantitative measurements of the spread and dissemination of the infection at regional and national levels. These findings contribute greatly to the fundamental understanding of the patterns and transmission dynamics of HIV during the armed conflict. The migration of HIV strains represents an enormous surveillance challenge. Hence, the national intervention policies during and at post-conflict periods should be implemented considering such understanding. Furthermore, geographically tracing interventions with specific incorporating strategies should be introduced, aiming to control HIV in Libya. Specific viral treatment therapy to the infected people should be introduced all over the country, specifically a national registry system for all infected patients so they could have the medical care that they may need.

## Data Availability Statement

The data presented in this paper are freely available upon request.

## Ethics Statement

Routinely collected sequence and demographic data on all newly notified HIV-1 infections are linked and irreversibly de-identified to enable public health research in the country as previously described (14, 16). The study was approved by the Libyan National Ethical Committee (Approval No. LY NS, HIV473221). It was conducted under the Helsinki Declaration and the supervision of the Libyan Study Group of Hepatitis & HIV (19, 20).

## Author Contributions

MAD conceived and designed the study, wrote the article, designed the analysis, analyzed the data, and performed the cartography. MAD and AE-B contributed to the analysis tools. AE-B and MA made substantial contributions to conception and design, acquisition of data, or analysis, and interpretation of data. MAD, MA, and AE-B provided advice and critically reviewed the manuscript. All authors read and approved the final manuscript.

## Conflict of Interest

The authors declare that the research was conducted in the absence of any commercial or financial relationships that could be construed as a potential conflict of interest.

## Publisher's Note

All claims expressed in this article are solely those of the authors and do not necessarily represent those of their affiliated organizations, or those of the publisher, the editors and the reviewers. Any product that may be evaluated in this article, or claim that may be made by its manufacturer, is not guaranteed or endorsed by the publisher.
